# Supplementary material for: Organisation of the orthobunyavirus tripodal spike and the structural changes induced by low pH and K+ during entry
Source: Nat Commun. 2023 Sep 21;14:5885. doi: 10.1038/s41467-023-41205-w (PMC10514341; doi:10.1038/s41467-023-41205-w)
Supplement: Supplementary file 1 — Supplementary Information [file 41467_2023_41205_MOESM1_ESM.pdf]

Supplementary Information

Organisation of the orthobunyavirus tripodal spike and the structural changes induced by low pH and K^+ during entry

Samantha Hover[1,2], Frank W Charlton[1,2], Jan Hellert[3], Jessica J Swanson[1,2], Jamel Mankouri[1,2]*, John N Barr[1,2]*, Juan Fontana[1,2]*

[1]School of Molecular and Cellular Biology, Faculty of Biological Sciences, [2]Astbury Centre for Structural and Molecular Biology, University of Leeds, LS2 9JT, United Kingdom.

[3]Centre for Structural Systems Biology, Leibniz-Institut für Virologie (LIV), Notkestraße 85, 22607 Hamburg, Germany.

*Correspondence: Dr Juan Fontana Tel: +44 (0)113 343 4170, Email: J.Fontana@leeds.ac.uk; Dr John N. Barr Tel: +44 (0)113 343 8069 Email: J.N.Barr@leeds.ac.uk; Dr Jamel Mankouri, Email: bms9jm@gmail.com

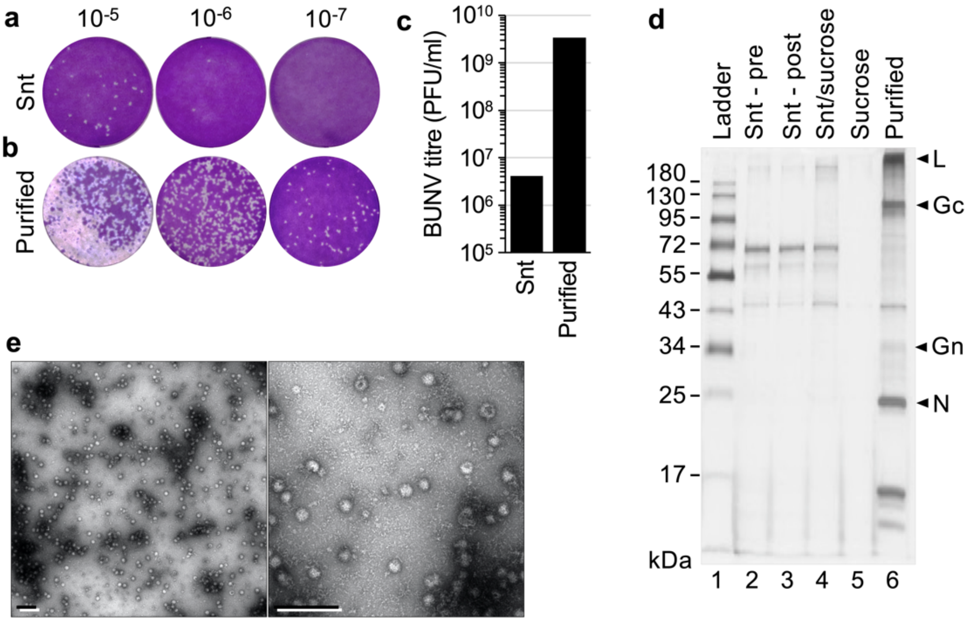

Supplementary Fig. 1 WT BUNV purification.

BHK-21 cells were infected with BUNV for 44 hrs and then viral supernatants (snt) were collected and purified by pelleting through a 30% sucrose cushion. Virus was resuspended in 0.1x PBS (purified) and **a,b,c** the titre was estimated by plaque assay as plaque forming units per ml (PFU/ml) (n=1). **d** SDS-PAGE followed by silver staining of purified BUNV alongside samples collected at each stage of purification; pre-ultracentrifugation supernatant (Snt – pre), post-ultracentrifugation supernatant (Snt – post), supernatant-sucrose cushion interface (Snt/sucrose), and sucrose cushion (Sucrose). The protein ladder sizes are indicated (kDa), as well as the bands corresponding to L, Gc, Gn and N (n=1). **e** Negative stain EM of purified BUNV loaded onto carbon coated EM grids and stained with 1% uranyl acetate. Scale bars = 500 nm (images are representative of 13 micrographs collected, n=2).

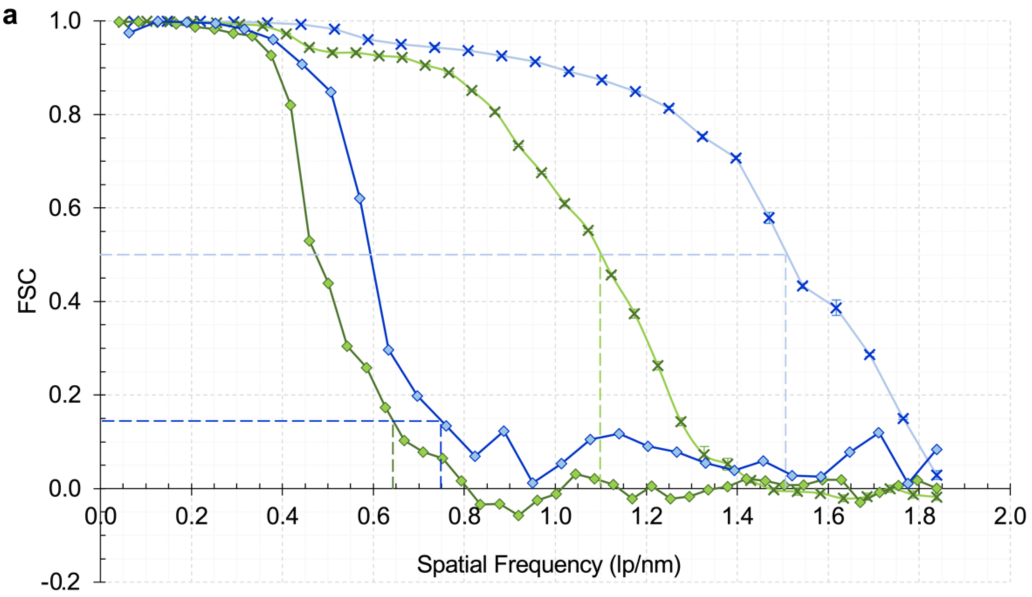

	FSC Calculation	FSC Cutoff	Spatial Freq (lp/nm)	Resolution (Å)
Tripod	Split UnbiasedFSC	0.143	0.65	15.5
	Full Dataset FSC	0.5	1.1	9.1
Floor	Split UnbiasedFSC	0.143	0.75	13.3
	Full Dataset FSC	0.5	1.51	6.6

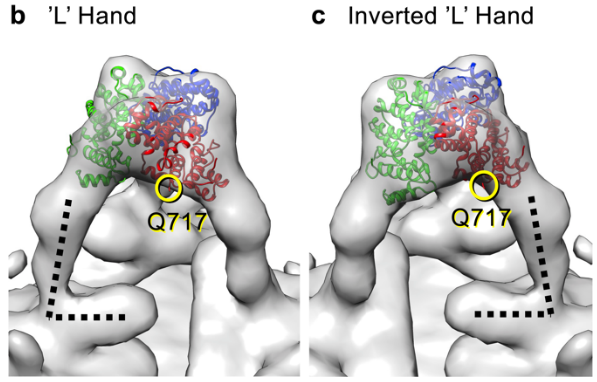

Supplementary Fig. 2 Further analysis of the BUNV GP STA.

a Fourier shell correlation (FSC) resolution (analysis of spatial frequency, line pairs per nm (lp/nm)) curves of the pH 7.3/no K^+ BUNV tripod (green) and floor (blue) STAs (Figs. 1,2). Gold-standard FSC (GS-FSC) calculations (♦ points) were performed by comparing the resolution of half datasets, using a 0.143 FSC cutoff to determine the resolution of each (Tripod STA = dark green line, Floor STA = dark blue line). Standard FSC calculations (× points) were additionally performed to determine the resolution of full datasets using a 0.5 cutoff (Tripod STA = light green line, Floor STA = light blue line). Error bars represent SD from the average of n=3 FSC calculations with different pseudo-random assortment of particles into half-sets using PEET. The resolution for each is indicated in angstroms (Å) and is determined as 1/spatial frequency in angstroms. **b,c** Fit of the BUNV head domain trimer (STA filtered bin=2 for comparison) when switching the handedness; b ‘L’ hand, c inverted ‘L’ hand. Q717 is the C-terminal residue of the head domain which connects to the stalk domain, and is more favourably localised in the inverted ‘L’ hand.

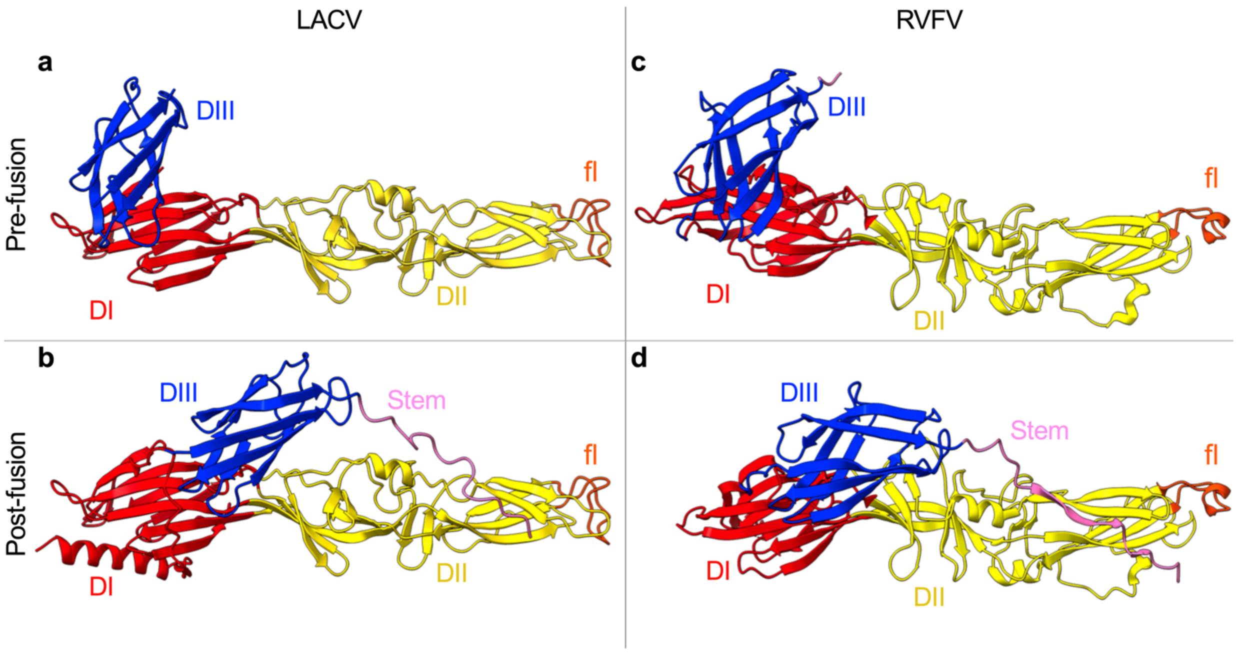

Supplementary Fig. 3 Comparison of the LACV and RVFV pre-fusion versus post-fusion class II fusion domain conformations.

A monomer of each conformation is represented, where Domain I (DI) is in red, domain II (DII) yellow, domain III (DIII) in blue, the fusion loop (fl) in DII is in orange, and the stem region (connecting to the TMD) is in pink. The **a** LACV pre-fusion fusion domain structure (residues 965-1344) is modelled from the **b** post-fusion LACV fusion domain structure (residues 928-1364; pdb: 7A57). DIII in *a* has been rotated from its position in b, based upon the canonical shift in DIII observed for class II fusion proteins in pre-fusion versus post-fusion conformations. RVFV is shown to exemplify the known shift in DIII during fusion events. RVFV fusion domain also forms a class II fold and structures have been solved for both the **c** pre-fusion (residues 688-1118; pdb: 4HJ1 (27)) and **d** post-fusion conformations (residues 691-1136; pdb: 6EGU (28)). The RVFV pre-fusion conformation (c) is represented as in the X-ray structure when Gc is expressed recombinantly outside the context of a virus (Fig. 4c represents RVFV Gc flexibly fitted within the EM average of the virus (7)).

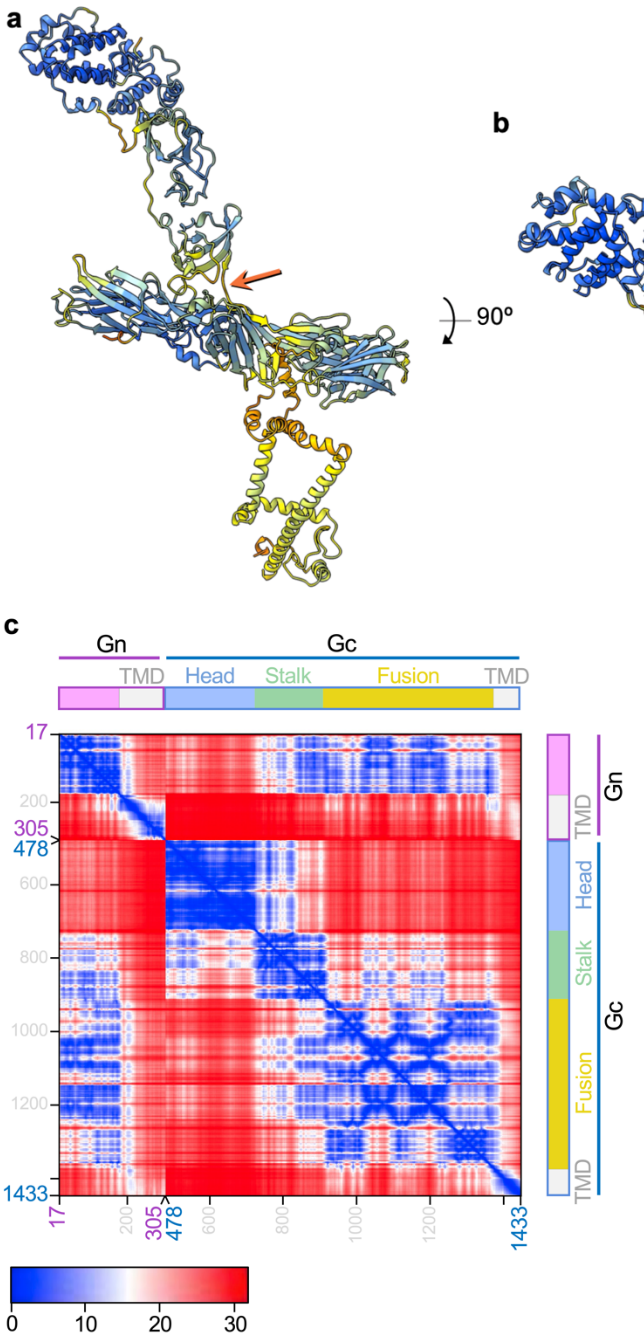

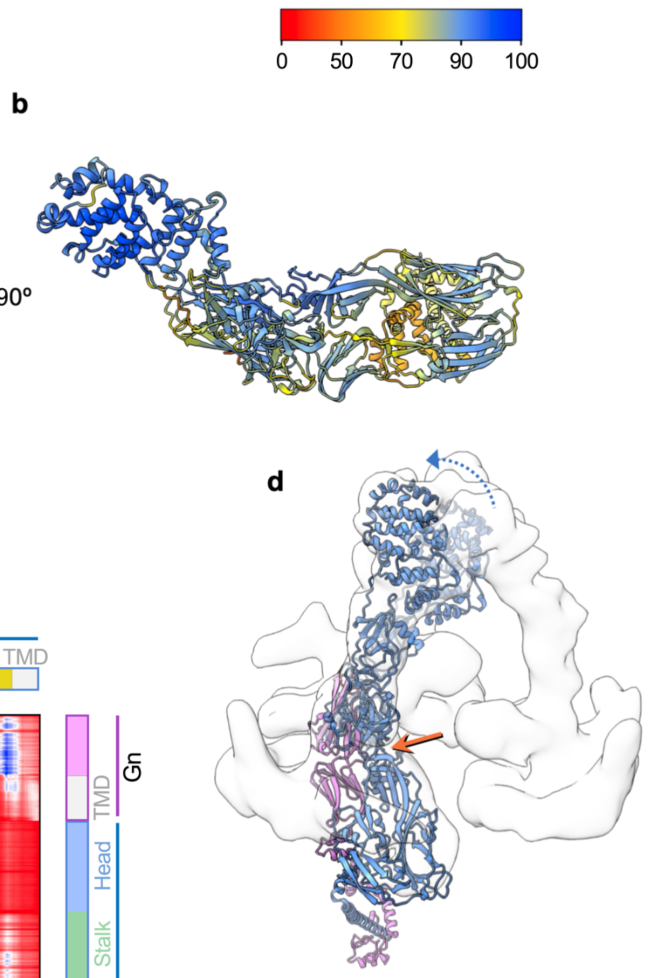

e

Gc domain	Virus species	PDB	AlphaFold model RMSD score (Å)
Head	BUNV	6H3V	0.419
Stalk	SBV	6H3S	1.087
Fusion DI	LACV	7A57	0.993
Fusion DII	LACV	7A57	0.867
Fusion DIII	LACV	7A57	0.930

Supplementary Fig. 4 AlphaFold modelling of BUNV Gn-Gc reveals the arrangement of the orthobunyavirus GP envelope.

a,b The BUNV Gn-Gc model generated by AlphaFold, as in Fig. 3a,b, coloured (ChimeraX AlphaFold palette) by the per-residue confidence score from 1-100 pLDDT (see colour key). The majority of residues have a confidence >70 (yellow to blue), indicating the residues were modelled well. Residues >90 are expected to be modelled to high accuracy (24). Key linker-regions have lower confidence (<50, orange) suggesting flexibility between domains compared to the generated model (orange arrow indicates rotation site for model fitting). **c** Predicted Aligned Error (PAE) plot of the AlphaFold model in a-b. The PAE measures the expected error in position between pairs of residues (x,y). A low score (blue) indicates the prediction has well-defined the relative position between pairs of residues in two domains (see colour key). A high score indicates uncertainty (Red). For each chain the numbers indicate the amino acid number in the polyprotein sequence. The key and labels indicate the regions which correspond to each protein and their sub-domains. **d** Comparison of the positions of the head and stalk domains in the generated AlphaFold model (translucent blue), with the fitted model rotated between the stalk and fusion domain interface (orange arrow indicates the residue about which the head-stalk were rotated, and the blue arrow the direction of rotation). Gc is coloured blue and Gn in pink. **d** A summary table of the RMSD scores (of pruned atom pairs) for the AlphaFold model with published structures of individual domains, as determined using the ChimeraX matchmaker function. The LACV Gc fusion sub-domains are provided individually as the published structure is in the post-fusion conformation.

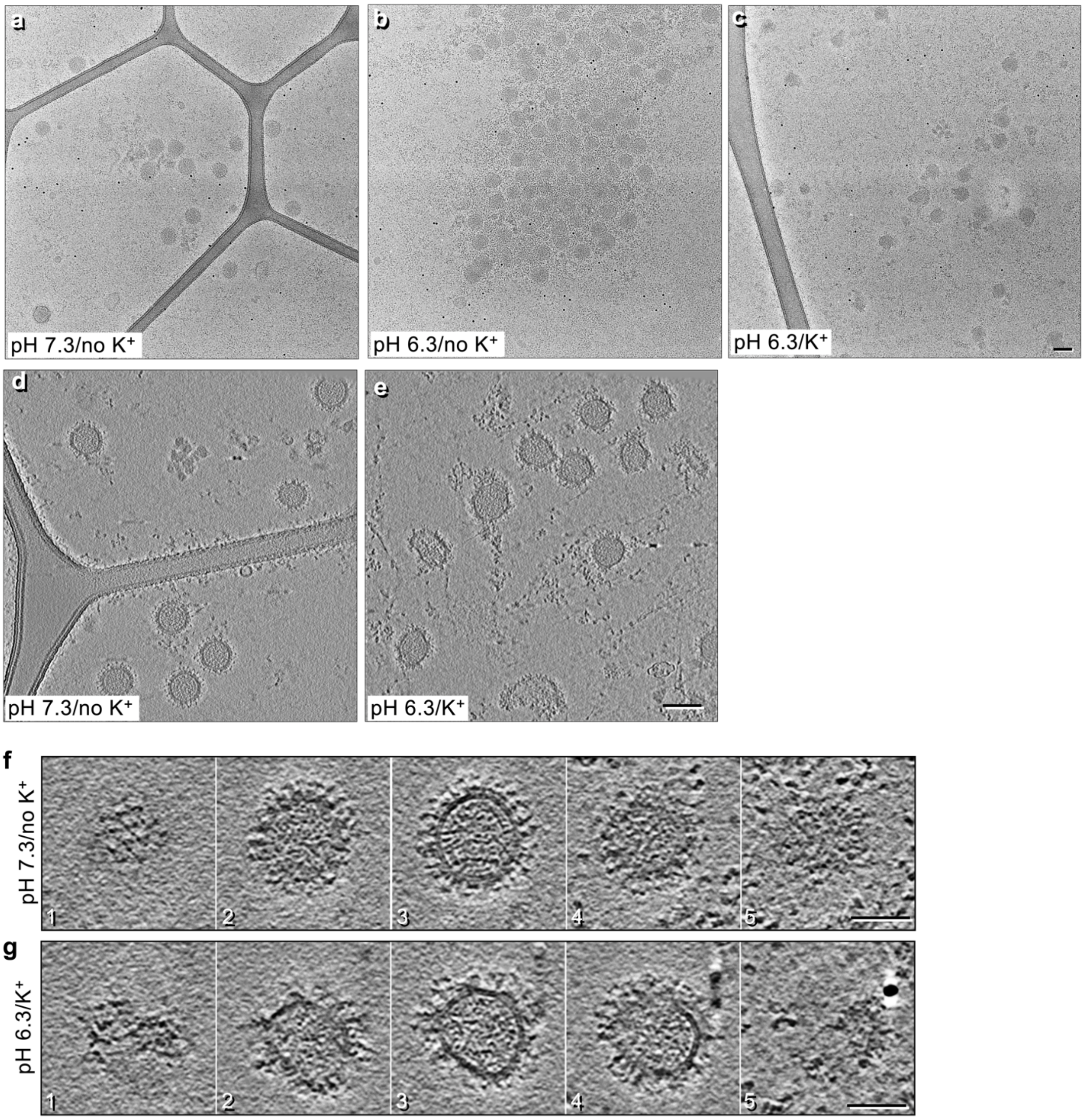

Supplementary Fig. 5 Cryo-EM and ET of BUNV virions under different pH and K^+ conditions.
Low magnification micrographs of images shown in Fig. 5a-c (n=2, >10 images per condition). **a** pH 7.3/no K^+, **b** pH 6.3/no K^+ and **c** pH 6.3/K^+ treated virions. **d,e** Cryo-ET slices through a **d** pH 7.3 and **e** pH 6.3/K^+ tomogram. **f,g** Slices through the z plane of the cryo-ET of individual pH 7.3/no K^+ (f) and pH 6.3/K^+ (g) virions (14 tomograms reconstructed per condition). Scale bars = 100 nm.

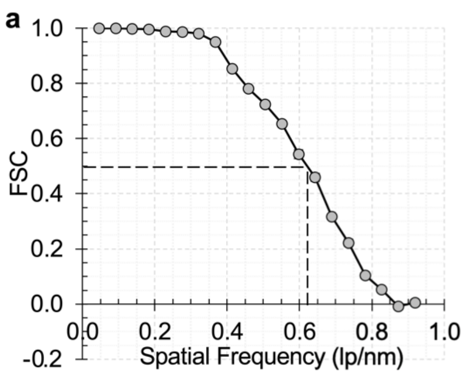

e

Domains	Species	PDB	CC score
Gc head/ stalk	BUNV/ SBV	6H3V/ 7A57	0.6742
Gc FD (post-fusion)	LACV	7A57	0.8097
Gn + Gc FD (pre-fusion)	BUNV	AlphaFold model	0.7396

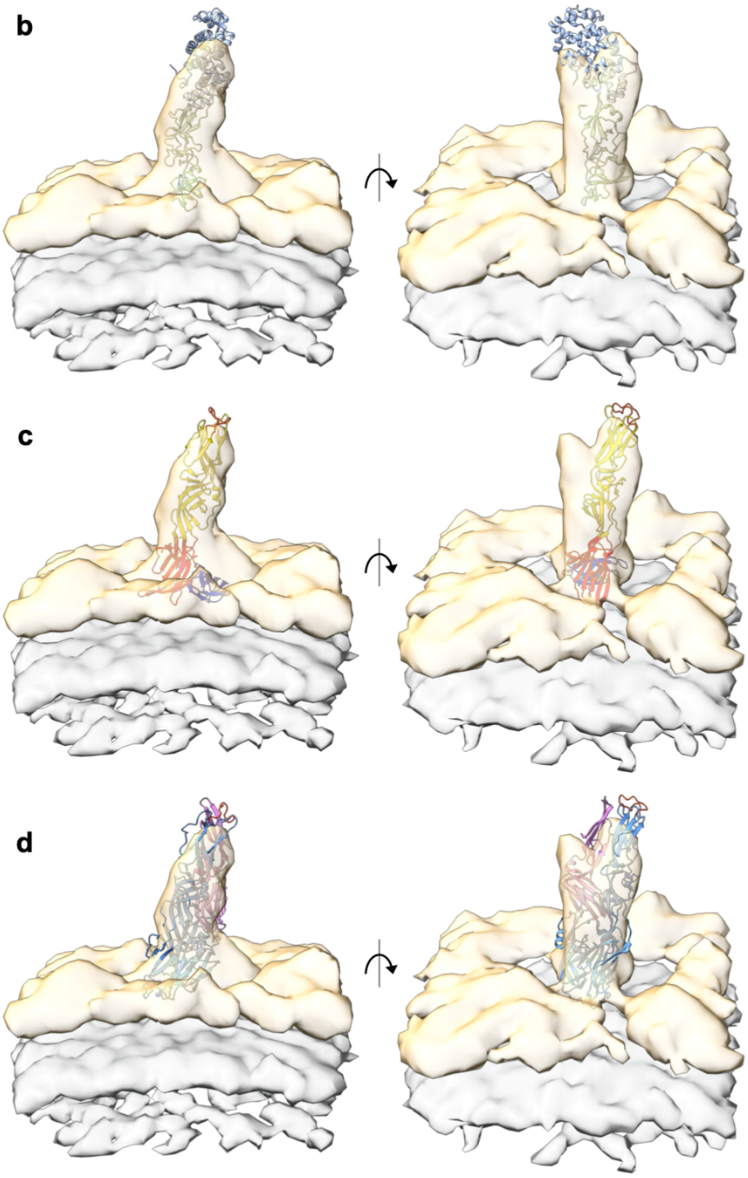

Supplementary Fig. 6 Fitting of solved orthobunyavirus Gc crystal structures on the pH 6.3/K^+ BUNV STA spike.

a Standard FSC resolution curve (as in Supplementary Fig. 2a) of the pH 6.3/K^+ BUNV STA (Fig. 5i,j). The resolution is ~16 Å, using a 0.5 cutoff (used owing to the flexibility of the structure). **b** The BUNV head (light blue, pdb: 6H3V) and SBV stalk (light green, pdb: 6H3S) domains fitted into the spike density. **c** The LACV fusion domain (domains I-III coloured as in Supplementary Fig. 3a) rotated perpendicular to the viral membrane (grey). DIII (dark blue) remains in proximity to the viral membrane (DIII connects to the C-terminal TMD), with the fusion loops (orange) extending away from the virus. **d** The AlphaFold model of Gn and the Gc fusion domain fitted within the spike. **e** Table summarising the cross-correlation (CC) score (0-1, where 1 indicates the best correlation) for each of the fitted models in b-d.

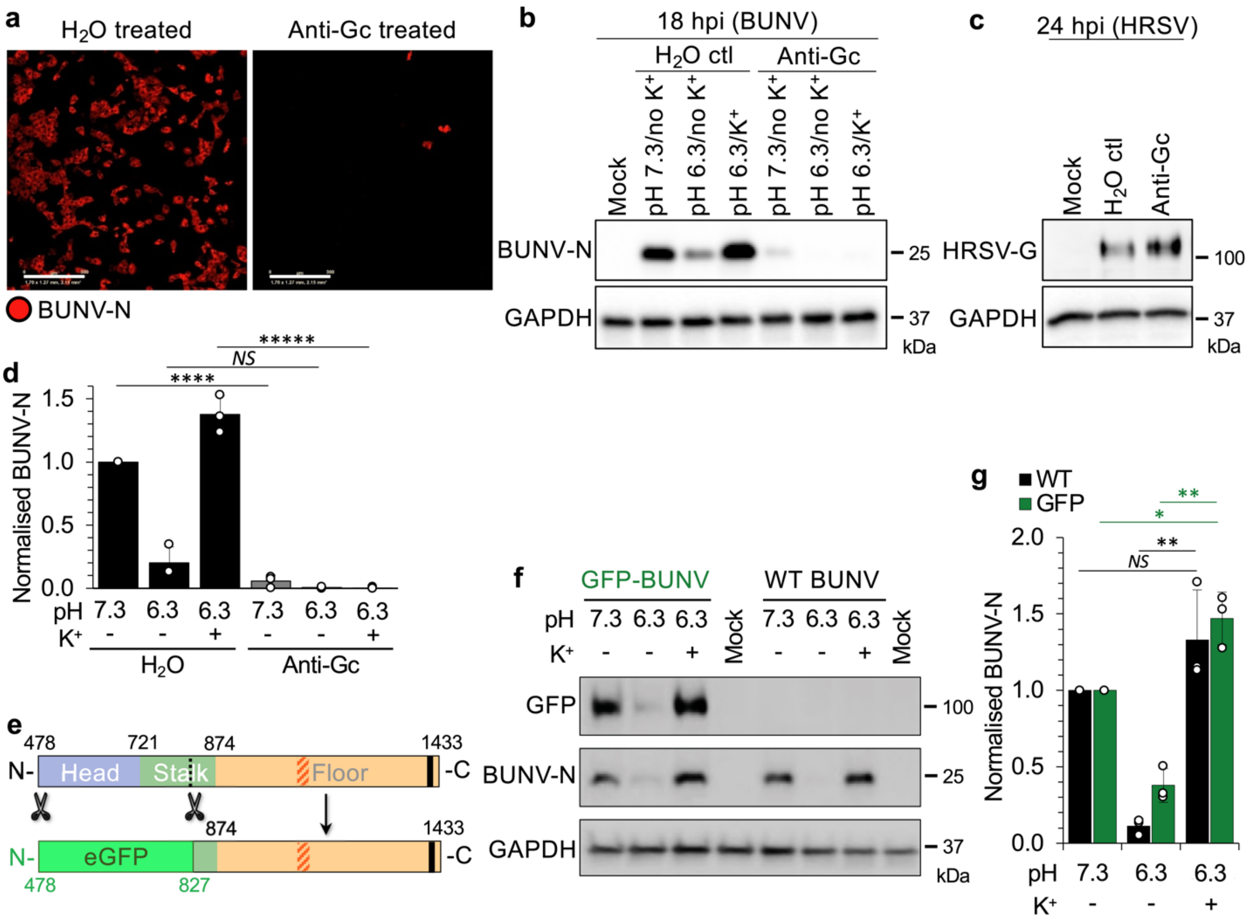

Supplementary Fig. 7 Biochemical characterisation of the Gc head domain after pH 6.3/K$^+$ treatment.

a BUNV virions were treated with dH_2O (control) or an anti-Gc neutralising antibody that binds the Gc head domain (mAb-742 (31)) for 1 hr. Virus was then added to A549 cells and incubated for 18 hrs, after which cells were fixed (18 hrs post-infection (hpi)), permeabilised and stained for anti BUNV-N with Alexa-Fluor-594 secondary antibodies. Fluorescence images were acquired using an IncuCyte Zoom, identifying fluorescent infected cells (n=2). **b** BUNV was *in vitro* treated for 2 hrs at 37˚C (as previously (19)), with pH 7.3/no K$^+$, pH 6.3/no K$^+$ or pH 6.3/K$^+$. Buffer was diluted out and virions neutralised with anti-Gc or a dH_2O control, and A549 cells infected as in a. An uninfected control, mock, was also included. Western blot analysis was performed as in Fig. 6b using anti-BUNV-N and anti-GAPDH (loading control) antibodies (n=3). The position of the nearest size markers are indicated (kDa). **c** As a control, HRSV was incubated with anti-Gc or dH_2O as in a and infection was assessed at 24 hpi using an anti-HRSV antibody, to confirm antibody specificity (n=3). **d** Densitometry of n=3 western blots from b normalised to each loading control and then to the pH 7.3/no K$^+$ H_2O-treated control (individual data points - white spheres). Error bars indicate mean ± SD (as in Fig. 6c), normalised to the 'H_2O' pH 7.3/no K$^+$ control. A one-way ANOVA was used to determine significance comparing each anti-Gc treated condition to the equivalent H_2O control: pH 7.3 P=4x10^{-6}, pH 6.3 P=8x10^{-5}, pH 6.3/K$^+$ was non-significant (NS) P=0.06. **e** Schematic of WT (coloured as in Fig. 1a) versus eGFP-tagged BUNV Gc protein (GFP-BUNV), with the removed section of the Gc head/stalk domains indicated, which were replaced with eGFP (green) (33). **f** WT and GFP-BUNV were treated with pH 7.3/no K$^+$, pH 6.3/no K$^+$ or pH 6.3/K$^+$, and infection (or non-infected, mock) was assessed by western blot at 18 hpi (n=3). **g** Densitometry of n=3 western blots from f (as in d). Black bars indicated WT BUNV and green GFP-BUNV. Statistical testing compared each WT or GFP virus condition to the corresponding pH 6.3/K$^+$ treated condition: pH 7.3 WT P=0.15 GFP P=0.009, pH 6.3 WT P=0.003 GFP P=0.0008.

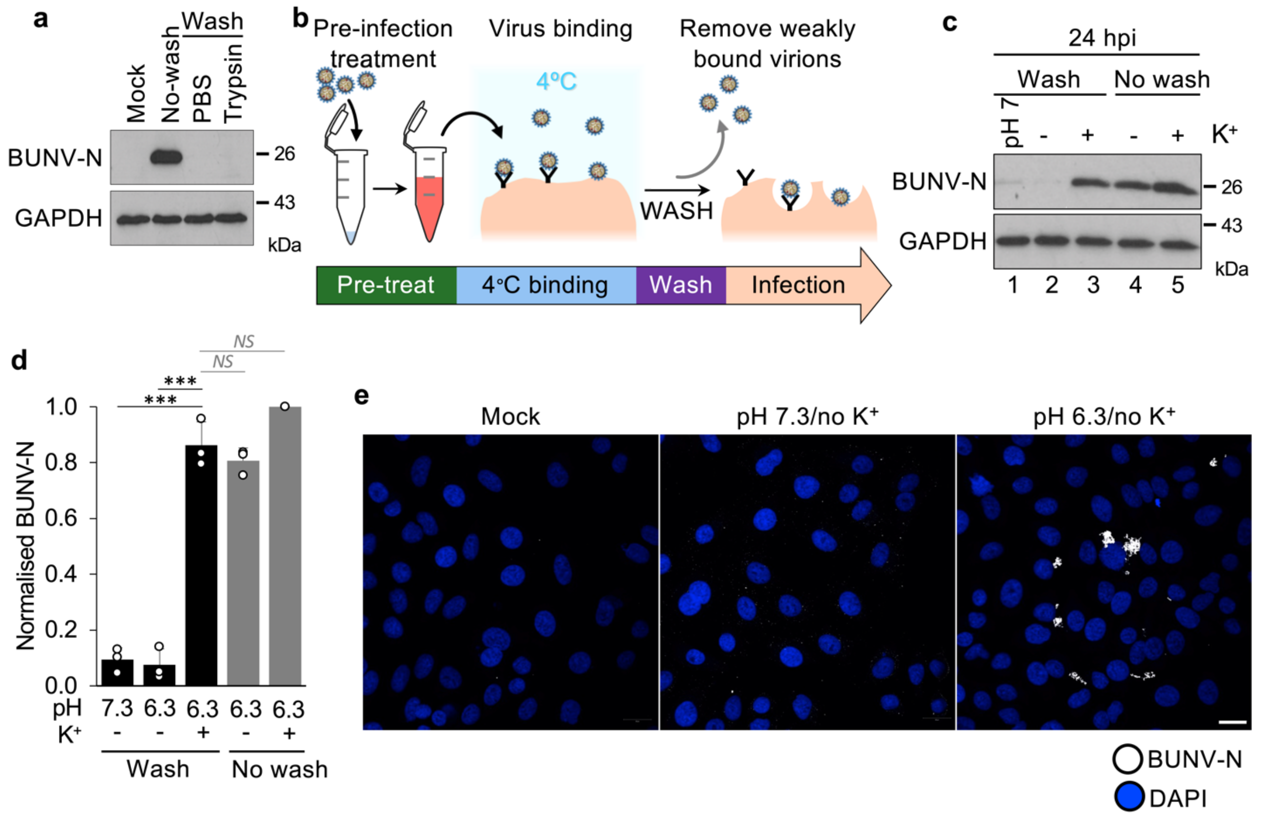

Supplementary Fig. 8 pH 6.3/K$^+$ *in vitro* treatment of BUNV subsequently increases interactions with host cells.

a Virions were bound to A549 cells at 4 ˚C and then weakly bound or unbound virions were removed with three PBS or 0.1x trypsin washes (or no-wash as a control). Cells were warmed to 37 ˚C and infected (or non-infected, 'mock') for 24 hrs. Western blot analysis of cell lysates (as in Fig. 6b) (n=3). **b** Workflow of the binding assay, whereby BUNV virions are *in vitro* treated for 2 hrs pre-infection with pH 7.3/no K$^+$ (as control, pH 7), pH 6.3/no K$^+$ (-) or pH 6.3/K$^+$ buffers (+) (as previously described (19)). Buffer is diluted out prior to virions being bound to cells at 4 ˚C. Weakly or unbound virions are removed by three PBS washes; or a no wash control. Infection is allowed to proceed for 24 hrs, then cells are lysed and **c** western blot analysed using antibodies against BUNV-N or GAPDH (n=3). **d** Densitometry of n=3 repeats of western blots in **c** (individual data points - white spheres), error bars indicate mean ± SD normalised to the no wash pH 6.3/K$^+$. Significant difference was determined using a one-way ANOVA, comparing each condition to the no wash pH 6.3/K$^+$: Wash pH 7.3 P=0.0001 pH 6.3 P=0.0002, No Wash were non-significant (NS) pH 6.3 P=0.37 pH 6.3/K$^+$ P=0.05. **e** Purified virions were *in vitro* treated with pH 7.3/no K$^+$ or pH 6.3/K$^+$ buffers, then an MOI = ~10 were bound to cells at 4 ˚C and then washed, similar to b-d, after which cells were fixed (0 hpi) using 4% paraformaldehyde and immunofluorescently labelled with anti-BUNV-N primary antibodies and Alexa-Fluor 488 nm secondary antibodies (white) to detect surface-bound virion clusters (n=3). Prolong gold with DAPI (blue) was used as a mounting reagent and images taken using a confocal microscope (n=3). Scale bar = 20 μm.

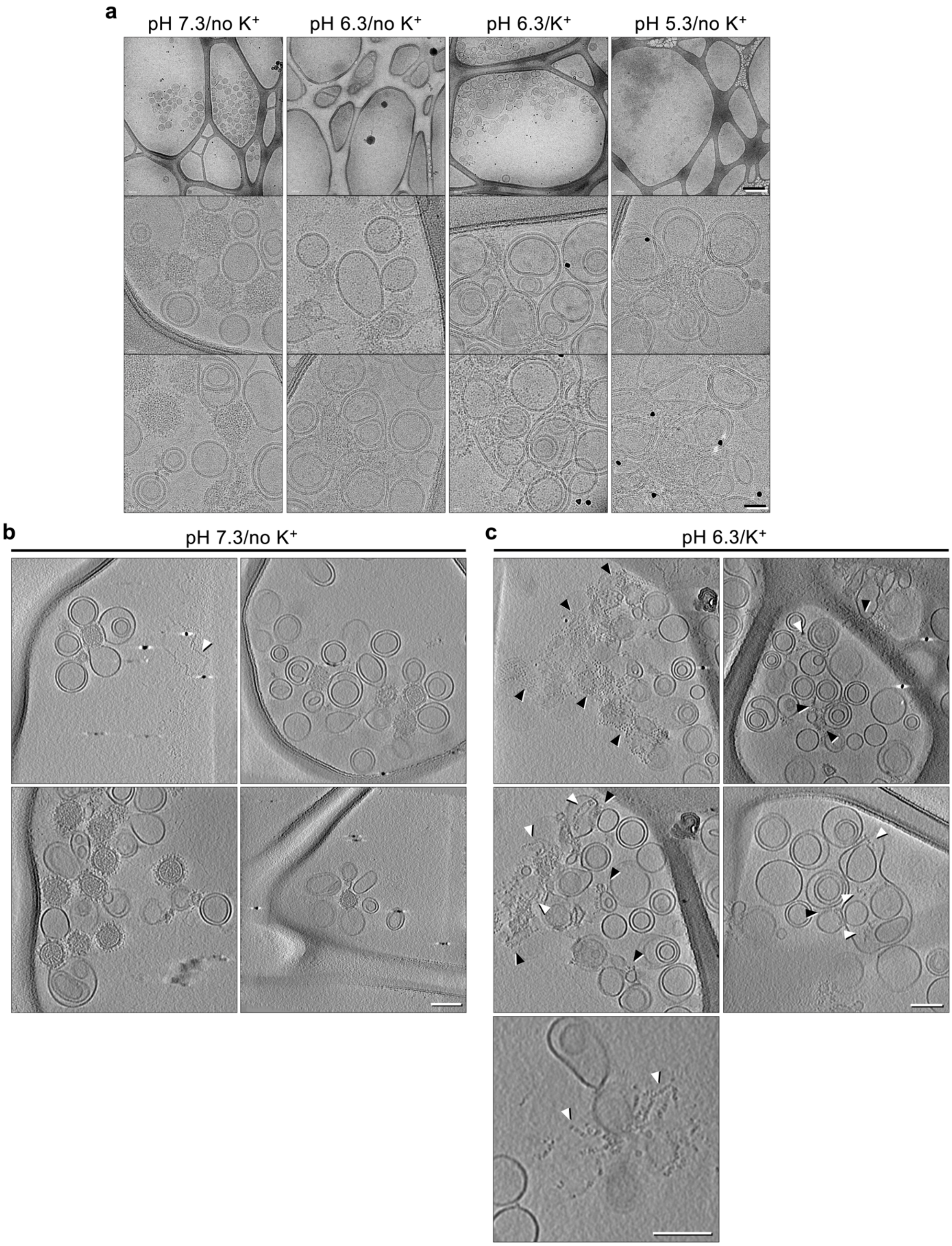
a
pH 7.3/no K+
pH 6.3/no K+
pH 6.3/K+
pH 5.3/no K+
b
pH 7.3/no K+
c
pH 6.3/K+

Supplementary Fig. 9 pH 6.3/K$^+$ induces fusion with target membranes.

a cryo-EM projections of the virus-liposome fusion assays from Fig. 6d-h (n=3, >30 images/condition). Conditions shown were virus-liposome mixtures incubated with pH 7.3/no K$^+$, pH 6.3/no K$^+$, pH 6.3/K$^+$ or pH 5.3/no K$^+$ buffers. **b,c** 3D tomographic sections of the **b** pH 7.3/no K$^+$ (6 tomograms) and **c** pH 6.3/K$^+$ conditions from Fig. 6d-h (9 tomograms per condition), showing additional tomograms (b 4 tomograms shown, c multiple planes through 4 tomograms shown) to exemplify features of the tomograms. In b, virions and liposomes are separate, while in c vRNPs can be found inside liposomes. White arrows indicate vRNPs and black arrows viral GPs. Scale bars in a low magnification images (top row) = 500 nm, higher magnification images (second and third rows) = 50 nm. Scale bars in b = 100 nm. A movie through a tomogram for each condition can be found as Supplementary Movies 3-4.

Supplementary Table 1 Cryo-ET and STA data processing summary.

	Tripod<br>pH 7.3	Floor<br>pH 7.3	pH 6.3/K^+
Tomograms	14		14
Viruses	71		89
Particle picking method	Automated – 'seedSpikes'		Manual selection
Full particles picked	8,912		19,634
Initial particles*	6,830		18,312
Average particles/virus	~96		~206
Symmetry	C3	C3	C1
Particles in class<br>(Number of classes)	N/A	N/A	10,349<br>(3)
Particles after C3 symmetry expansion	21,498	16,368	N/A
Final particles<br>(split dataset particles)	20,282<br>(11,754)	16,096<br>(10,330)	9,987
Pixel size (Å)	2.72	2.72	2.72
Split dataset resolution, cutoff 0.143 (Å)	15.5	13.3	N/A
Full dataset resolution, cutoff 0.5 (Å)	9.1	6.6	16
AlphaFold Gn-Gc dimer cross-correlation score	0.7214	0.7328	N/A
EMDB deposition code	EMD-15557	EMD-15569	EMD-15579

* Total particles after an initial iteration to remove duplicates
