# Supplementary material for: Organisation of the orthobunyavirus tripodal spike and the structural changes induced by low pH and K+ during entry
Source: Nat Commun. 2023 Sep 21;14:5885. doi: 10.1038/s41467-023-41205-w (PMC10514341; doi:10.1038/s41467-023-41205-w)
Supplement: Supplementary file 3 — Description of Additional Supplementary Files [file 41467_2023_41205_MOESM3_ESM.pdf]

Description of Additional Supplementary Files

File Name: Supplementary Data 1
Description: BUNV Gn and Gc ectodomain AlphaFold prediction as is (rigid-body) fitted within the BUNV tripod STA (Fig. 3c,d); TMDs removed as they do not comply with the STA density (represented are residues Gn 17-205, Gc 478-1382).

File Name: Supplementary Movie 1
Description: Fit of available orthobunyavirus partial-Gc structures and of the BUNV Gn-Gc AlphaFold model within the tripod and floor STA. The BUNV head (6H3V), SBV stalk (6H3S) and LACV fusion domain are fitted as in Figs. 1,2; and the BUNV AlphaFold as in Fig. 3. CL = capping loop on Gn, FL = fusion loop on Gc. The floor STA is also compared with the pH 6.3/K+ virus 'spike' as in Fig 5i,j.

File Name: Supplementary Movie 2
Description: Modelling the BUNV envelope. An AlphaFold model of the BUNV GP envelope is predicted, showing three hetero-hexamers fitted within both the tripod and the floor region STA as in Fig. 3g,h. The wider lattice-like model of the viral envelope can be inferred from these arrangements. The clashes between some of the head domains from opposing Gc monomers are potentially due to flexibility between the stalk sub-domains, across the local lattice arrangement, and due to the curvature of the viral membrane; which are not accounted for in this model.

File Name: Supplementary Movie 3
Description: Liposome fusion assay with BUNV at pH 7.3/no K+. A movie through a tomogram from the fusion assay from Fig. 6d and Supplementary Fig. 9b.

File Name: Supplementary Movie 4
Description: Liposome fusion assay with BUNV at pH 6.3/K+. A movie through a tomogram from the fusion assay from Fig. 6g,h (matching the segmentation tomogram) and Supplementary Fig. 9c.
