# Supplementary material for: Organisation of the orthobunyavirus tripodal spike and the structural changes induced by low pH and K+ during entry
Source: Nat Commun. 2023 Sep 21;14:5885. doi: 10.1038/s41467-023-41205-w (PMC10514341; doi:10.1038/s41467-023-41205-w)
Supplement: Supplementary file 9 — Reporting Summary [file 41467_2023_41205_MOESM9_ESM.pdf]

nature portfolio

Corresponding author(s): Juan Fontana

Last updated by author(s): Aug 11, 2023

Reporting Summary

Nature Portfolio wishes to improve the reproducibility of the work that we publish. This form provides structure for consistency and transparency in reporting. For further information on Nature Portfolio policies, see our Editorial Policies and the Editorial Policy Checklist.

Statistics

For all statistical analyses, confirm that the following items are present in the figure legend, table legend, main text, or Methods section.

n/a	Confirmed	
☐	☒	The exact sample size (*n*) for each experimental group/condition, given as a discrete number and unit of measurement
☐	☒	A statement on whether measurements were taken from distinct samples or whether the same sample was measured repeatedly
☐	☒	The statistical test(s) used AND whether they are one- or two-sided<br>*Only common tests should be described solely by name; describe more complex techniques in the Methods section.*
☒	☐	A description of all covariates tested
☒	☐	A description of any assumptions or corrections, such as tests of normality and adjustment for multiple comparisons
☐	☒	A full description of the statistical parameters including central tendency (e.g. means) or other basic estimates (e.g. regression coefficient) AND variation (e.g. standard deviation) or associated estimates of uncertainty (e.g. confidence intervals)
☐	☒	For null hypothesis testing, the test statistic (e.g. *F*, *t*, *r*) with confidence intervals, effect sizes, degrees of freedom and *P* value noted<br>*Give P values as exact values whenever suitable.*
☒	☐	For Bayesian analysis, information on the choice of priors and Markov chain Monte Carlo settings
☒	☐	For hierarchical and complex designs, identification of the appropriate level for tests and full reporting of outcomes
☒	☐	Estimates of effect sizes (e.g. Cohen's *d*, Pearson's *r*), indicating how they were calculated

Our web collection on statistics for biologists contains articles on many of the points above.

Software and code

Policy information about availability of computer code

Data collection

For BUNV viral particle cryo-ET data collection Tomography v4 and EPU v2.8.1 were used.
For liposome-virus fusion assay cryo-ET data was collected using TOMO v5.13.

Data analysis

Tomogram motion correction and gctf calculation:
Relion v3.0.0

Tomogram reconstruction:
IMOD v4.9.11

Sub-tomogram averaging:
PEET v1.14.0
Bsoft v2.0.4

Tomogram and average visualisation & western blot image analysis:
Fiji ImageJ v2.1.0

Tomogram Segmentation:
AmiraEM v6.5.0 (Thermo Scientific)

Structural analysis, modelling and representation:

AlphaFold_multimer v2.1.0 (as a local install as per https://github.com/deepmind/alphafold)
Coot 0.9.6
UCSF Chimera 1.11.2
UCSF ChimeraX 1.5

Statistical analysis:
Microsoft Excel v16.66.1

For manuscripts utilizing custom algorithms or software that are central to the research but not yet described in published literature, software must be made available to editors and reviewers. We strongly encourage code deposition in a community repository (e.g. GitHub). See the Nature Portfolio guidelines for submitting code & software for further information.

Data

Policy information about availability of data

All manuscripts must include a data availability statement. This statement should provide the following information, where applicable:

- Accession codes, unique identifiers, or web links for publicly available datasets
- A description of any restrictions on data availability
- For clinical datasets or third party data, please ensure that the statement adheres to our policy

The cryo-ET STA averages have been deposited in EMBD under accession codes EMD-15557 [https://www.ebi.ac.uk/emdb/EMD-15557] (STA of the pH 7.3/no K+ BUNV tripod in Fig. 1); EMD-15569 [https://www.ebi.ac.uk/emdb/EMD-15569] (STA of the floor region in Fig. 2); and EMD-15579 [https://www.ebi.ac.uk/emdb/EMD-15579] (STA of the pH 6.3/K+ GPs in Fig. 5). The AlphaFold model (Fig. 3), lacking the TMDs (which are not supported by the STA data) can be found as Supplementary Data 1.
The previously published X-ray crystal structures can be obtained from PDB using accession codes 6H3V [https://doi.org/10.2210/pdb6H3V/pdb] (BUNV Gc head domain); 6H3S [https://doi.org/10.2210/pdb6H3S/pdb] (SBV Gc head/stalk domains); and 7A57 [https://doi.org/10.2210/pdb7A57/pdb] (LACV Gc fusion domains in the post-fusion conformation).

Human research participants

Policy information about studies involving human research participants and Sex and Gender in Research.

Reporting on sex and gender	No human research participants were used in this study
Population characteristics	No human research participants were used in this study
Recruitment	No human research participants were used in this study
Ethics oversight	No human research participants were used in this study

Note that full information on the approval of the study protocol must also be provided in the manuscript.

Field-specific reporting

Please select the one below that is the best fit for your research. If you are not sure, read the appropriate sections before making your selection.

☒ Life sciences ☐ Behavioural & social sciences ☐ Ecological, evolutionary & environmental sciences

For a reference copy of the document with all sections, see nature.com/documents/nr-reporting-summary-flat.pdf

Life sciences study design

All studies must disclose on these points even when the disclosure is negative.

Sample size	Cryo-EM data collection and tomograms were processed from the best grids and data sets collected after extensive optimisation of sample and grid preparation methods.
Data exclusions	No data were excluded.
Replication	For all biomolecular assays, at least three independent experimental replicates were performed (n=3) to get the same result in all replicates, as is standard practice in the field.<br>For the STA, the datasets are separated into two at the start of processing and aligned independently. This allows for more accurate resolution determination by comparison of the averages at the end.<br>AlphaFold multimer to generate the BUNV Gn-Gc model performed 5 iterations before generating the final 5 models. The model ranked highest in terms of pLDDT score was used for fitting into STA.

Randomization	Our data does not require randomisation
Blinding	No blinding experiments are included. An individual performed each experiment based upon expertise.

Reporting for specific materials, systems and methods

We require information from authors about some types of materials, experimental systems and methods used in many studies. Here, indicate whether each material, system or method listed is relevant to your study. If you are not sure if a list item applies to your research, read the appropriate section before selecting a response.

Materials & experimental systems

n/a	Involved in the study
☐	☒ Antibodies
☐	☒ Eukaryotic cell lines
☒	☐ Palaeontology and archaeology
☒	☐ Animals and other organisms
☒	☐ Clinical data
☒	☐ Dual use research of concern

Methods

n/a	Involved in the study
☒	☐ ChIP-seq
☒	☐ Flow cytometry
☒	☐ MRI-based neuroimaging

Antibodies

Antibodies used

Primary Antibodies:
Anti-BUNV-N, sheep (J.N. Barr; University of Leeds, Leeds, UK) western blot dilution 1:5000 in 5% milk in TBS-T, immunofluorescence dilution 1:5000 in 1% BSA in PBS – antibodies were generated previously by Antibody Applications Ltd; serum was collected from a sheep after immunising with BUNV-N protein, which had been expressed and purified within the Barr lab (Ariza et al. (2013) NAR, reference 44).
Anti-BUNV-Gc mAb-742, mouse (Xiaohong Shi; Prof. Richard Elliott lab, Centre for Virus Research, University of Glasgow, Glasgow, UK) neutralisation dilution 1:10,000 in PBS. Monoclonal antibodies against BUNV were generated previously by Lappin et al. (1994) J. Virol (reference 30) - mice were inoculated with a BUNV-infected mouse cell lysate and boosted with BUNV. A MAb against Gc was identified, characterised, and validated.
Anti-HRSV, goat (Abcam ab20745) western blot dilution 1:1000 in 5% milk in TBS-T.
Anti-GAPDH, mouse (Santa Cruz sc47724) western blot dilution 1:1000 in 5% milk in TBS-T.
Anti-GFP, mouse (Santa Cruz sc9996) western blot dilution 1:1000 in 5% milk in TBS-T.

Secondary Antibodies:
Anti-sheep HRP-conjugated (Merck A3415) western blot dilution 1:5000 in 5% milk in TBS-T.
Anti-goat HRP-conjugated (Merck A8919) western blot dilution 1:5000 in 5% milk in TBS-T.
Anti-mouse HRP-conjugated (Merck A4416) western blot dilution 1:5000 in 5% milk in TBS-T.
Anti-sheep Alexa-fluor-488 conjugated (Thermo Scientific A11015) immunofluorescence dilution 1:500 in 1% BSA in PBS.
Anti-sheep Alexa-fluor-594 conjugated (Thermo Scientific A11016) immunofluorescence dilution 1:500 in 1% BSA in PBS.

Validation

Anti-BUNV-N antibodies have been extensively tested by western blot and immunofluorescence, comparing infected cells to uninfected controls across multiple cell lines. These data have previously been reported in multiple publications including: Ariza et al. (2013) NAR, Hover et al. (2016) JBC, Hover et al. (2018) Plos Path, Charlton et al. (2019) JBC, Hopkins et al. (2022) mBio.
Anti-BUNV-Gc Mab-742 antibodies were previously validated by Lappin et al. (1994) J.Virol, by immunoprecipitation and immunofluorescence. They have since been used in a number of publications including: Weber et al. (2001) Virology, Shi et al. (2004) J. Virol, Shi et al. (2005) J. Virol, Shi et al. (2006) J. Virol, Shi et al. (2007) J. Virol, Shi et al. (2010) J. Virol, Sanz-Sanchez and Risco (2013) Plos One. In this neutralisation assay we confirmed specificity to BUNV by the lack of neutralising activity against HRSV.

Eukaryotic cell lines

Policy information about cell lines and Sex and Gender in Research

Cell line source(s)	All cell lines were obtained from the European Collection of Cell Cultures (ECACC):<br>A549 human alveolar carcinoma epithelial cells (86012804)<br>BHK 21 baby hamster kidney cells (85011433)<br>SW 13 human adrenal cortex carcinoma cells (87031801)
Authentication	Authentication was performed by ECACC and not in-house.
Mycoplasma contamination	Cells are routinely tested for mycoplasma contamination.
Commonly misidentified lines<br>(See ICLAC register)	No commonly misidentified cell lines were used in this study.
